# Brain Network Topology in Deficit and Non-Deficit Schizophrenia: Application of Graph Theory to Local and Global Indices

**DOI:** 10.3390/jpm13050799

**Published:** 2023-05-06

**Authors:** Daniela Vecchio, Fabrizio Piras, Valentina Ciullo, Federica Piras, Federica Natalizi, Giuseppe Ducci, Sonia Ambrogi, Gianfranco Spalletta, Nerisa Banaj

**Affiliations:** 1 Laboratory of Neuropsychiatry, Department of Clinical and Behavioral Neurology, IRCCS Santa Lucia Foundation, 00179 Rome, Italy; d.vecchio@hsantalucia.it (D.V.);; 2 Department of Psychology, “Sapienza” University of Rome, Via dei Marsi 78, 00185 Rome, Italy; 3 PhD Program in Behavioral Neuroscience, Sapienza University of Rome, 00161 Rome, Italy; 4 Department of Mental Health, ASL Roma 1, 00135 Rome, Italy; 5 Menninger Department of Psychiatry and Behavioral Sciences, Baylor College of Medicine, Houston, TX 77030, USA

**Keywords:** neuroimaging, clinical neuroscience, brain structural covariance, mental diseases, psychiatric disorders

## Abstract

Patients with deficit schizophrenia (SZD) suffer from primary and enduring negative symptoms. Limited pieces of evidence and neuroimaging studies indicate they differ from patients with non-deficit schizophrenia (SZND) in neurobiological aspects, but the results are far from conclusive. We applied for the first time, graph theory analyses to discriminate local and global indices of brain network topology in SZD and SZND patients compared with healthy controls (HC). High-resolution T1-weighted images were acquired for 21 SZD patients, 21 SZND patients, and 21 HC to measure cortical thickness from 68 brain regions. Graph-based metrics (i.e., centrality, segregation, and integration) were computed and compared among groups, at both global and regional networks. When compared to HC, at the regional level, SZND were characterized by temporoparietal segregation and integration differences, while SZD showed widespread alterations in all network measures. SZD also showed less segregated network topology at the global level in comparison to HC. SZD and SZND differed in terms of centrality and integration measures in nodes belonging to the left temporoparietal cortex and to the limbic system. SZD is characterized by topological features in the network architecture of brain regions involved in negative symptomatology. Such results help to better define the neurobiology of SZD (SZD: Deficit Schizophrenia; SZND: Non-Deficit Schizophrenia; SZ: Schizophrenia; HC: healthy controls; CC: clustering coefficient; L: characteristic path length; E: efficiency; D: degree; CC_node_: CC of a node; CC_glob_: the global CC of the network; E_loc_: efficiency of the information transfer flow either within segregated subgraphs or neighborhoods nodes; E_glob_: efficiency of the information transfer flow among the global network; FDA: Functional Data Analysis; and D_min_: estimated minimum densities).

## 1. Introduction

The clinical heterogeneity of schizophrenia (SZ) has widely captured the interest of researchers and clinicians worldwide. A large amount of neuroanatomical, neurobiological, and neuropsychological research has been conducted with the aim of discriminating between subtypes of schizophrenia characterized by more homogeneous symptom domains [1]. In this view, patients with SZ suffering from primary, stable, and enduring negative symptoms, have been considered as a separate nosological entity, namely deficit schizophrenia (SZD) [2,3,4,5,6]. However, SZD cannot be merely considered the extreme end of a continuum. Indeed, evidence supports the hypothesis that compared with patients in the non-deficit subgroup (SZND), SZD is a separate disorder rather than a more severe form of SZ [3,7,8,9,10,11].

Thinner cortical thickness is a consistent finding in schizophrenia (SZ), as revealed by several studies [12,13,14,15,16] reporting irregularly distributed reductions across multiple loci. Most of the investigations compared diagnostic groups with mass univariate analysis and revealed localized regional changes, in a segregationist view [12,13,14,15]. However, this approach failed to quantify changes in the inter-relationship among different brain areas. Indeed, the brain is an integrated network emerging from a collective development, and a substantial body of evidence supports the hypothesis that SZ is a developmental disorder in which disruptions of cerebral connectivity and morphology contribute to the emergence of symptoms [17].

Essential information about abnormalities in brain development can be gathered by studying the structural covariance between brain regions morphology. Indeed, morphological networks, based on anatomical covariance among brain regions, identify an important aspect of developmental maturation, crucial to understand the pathophysiology of psychotic disorders [18,19]. Direct evidence linking anatomical covariance among brain regions to coordinated physiological brain development was described in recent studies [18,20,21]. Graph theory offers a promising technique for investigating the organization of the pairwise connections (covariance) between nodes of such brain networks [22]. Application of graph theory to neuroimaging data revealed that in the uninjured human brain, regions tend to be connected creating an efficient ‘small world’ network. This means that key brain regions are connected to multiple nearby brain regions in a modular or segregated fashion and are linked by short paths (traveling for long-range connections) that integrate such modules [23].

Several studies have already supported the evidence of altered brain network topology and structural covariance in SZ [24,25]. Specifically, the pattern of connections generally reveals a more segregated, less integrated, and inefficient brain system based on volumetric [26], thickness [16], gyrification [27], microstructural [28], and functional connectivity [25,28,29] measures. In these studies, the sources of heterogeneity are multifactorial, ranging from the MRI technique to the brain- or graph-based measures employed for analyses, leading to inconsistent results. Moreover, the much-discussed source of inconsistency in SZ results may be related to the substantial clinical heterogeneity, also associated with its multiple pathogenetic mechanisms, which Bleuler in the early years of the last century already labeled properly as a “Group of Schizophrenias” [30]. Support for clinical variability in SZ [31] comes from more recent studies that used unsupervised machine learning [32] or factorial [33] approaches and generated fascinating evidence for discrete categories of the disorder. Thus, a powerful approach to study SZ relies on separating more homogeneous subgroups of patients, in order to better characterize such disabling mental disorders.

Graph theory analysis of cortical thickness data may be used to highlight structural abnormalities in SZD at the network level. Although some studies suggested regional cortical thinning in SZD compared with SZND [34], others failed to reveal differences [35]. In this scenario, all studies have been conducted using univariate mass-based approaches, thus possibly overlooking potential differences in cortical thickness covariance between diagnostic groups.

This work aimed to investigate the hypothesis that different patterns of brain network abnormalities characterize SZD and SZND, and to contrast them with healthy controls (HC). To investigate our hypothesis, we compared the three groups with respect to global and regional brain network indices (described in Section 2.5) calculated on thickness-based measures.

## 2. Material and Methods

### 2.1. Study Sample

One hundred and fifteen patients diagnosed with SZ were initially assessed at IRCCS Santa Lucia Foundation in Rome, between March 2016 and May 2019. Clinicians who had treated the patients and knew their clinical history made the preliminary diagnosis. Then, a senior research psychiatrist (G.S.) confirmed all preliminary diagnoses using the Structured Clinical Interview for the DSM-5 Research Version (SCID-5 for DSM-5, Research Version; SCID-5-RV) [36]. Overall symptom severity of SZ was assessed using the Scale for the Assessment of Positive Symptoms (SAPS) [37] and the Scale for the Assessment of Negative Symptoms (SANS) [38], while the characterization of SZ subtype was done according to the Schedule for the Deficit Syndrome (SDS) [39] to distinguish SZD and SZND groups.

Five patients were excluded because they were not classifiable as SZD or SZND according to the SDS criteria and six patients because they were unable to complete the MRI examination, or because of the presence of MRI artifacts or brain abnormalities. Twenty-one out of the remaining 104 patients were classified as SZD while 83 were identified as SZND. We reduced the SZND group to 21 patients closely matched one by one for age (±2 years) and sex to the SZD ones, in order to obtain a comparable sample size. This was done to prevent correlational matrices and structural covariance measures from being biased by differences in patients’ sample size. Finally, 21 HC were recruited through local advertising and closely matched one by one for age (±2 years) and sex to the SZ subtype samples. All HC were screened for a current or lifetime history of DSM-5 psychiatric and personality disorders using the SCID-5-RV [36] and SCID-5-PD [40]. They were also assessed to confirm that no first-degree relative had a history of psychosis.

Inclusion criteria for all subjects were as follows: (i) age between 18 and 65 years, (ii) at least 8 years of education, and (iii) suitability for MRI scanning. Exclusion criteria were as follows: (i) history of alcohol or drug abuse in the 2 years prior to assessment, (ii) lifetime drug dependence, (iii) traumatic head injury with loss of consciousness, (iv) past or present major medical illness or neurological disorders, (v) any (for HC) or additional (for SZ) psychiatric disorder or mental retardation, (vi) dementia or cognitive deterioration according to DSM-5 criteria, and a mini-mental state examination (MMSE) [41] score <25, consistent with normative data in the Italian population [42], (vii) low T1-weighted images quality (i.e., presence of significant motion or scanner-generated artifacts), (viii) any brain abnormalities or microvascular lesions as apparent on conventional T2-weighted or fluid-attenuated inversion recovery (FLAIR) scans, potentially explaining the critical phenomenology. The extent of vascular lesions was assessed using the semi-automated method developed by our group [43].

Sociodemographic characteristics, SAPS and SANS scores, duration of illness, and antipsychotic dosages (in chlorpromazine equivalents) are summarized in Table 1.

The study was approved and undertaken in accordance with the guidelines of the Santa Lucia Foundation Ethics Committee. All participants gave written informed consent to participate after receiving a full explanation of the study procedures.

### 2.2. Image Acquisition E Processing

Each participant underwent the same imaging protocol, which included T2-weighted, FLAIR, and whole-brain 3D high-resolution T1-weighted sequences using a 3T Allegra MR imager (Siemens, Erlangen, Germany) with a standard quadrature head coil. Whole-brain 3D T1-weighted images were obtained in the sagittal plane using a modified driven equilibrium Fourier transform (MDEFT) sequence (TE/TR = 2.4/7.92 ms, flip-angle 15°, voxel-size 1 × 1 × 1 mm^3^) [44]. T2-weighted and FLAIR sequences were acquired to screen for brain pathology.

### 2.3. Cortical Thickness Estimation

To estimate cortical thickness, all whole-brain 3D T1-weighted images were processed using the automated and validated “recon-all” pipeline, as implemented in FreeSurfer (version 5.3.) (https://surfer.nmr.mgh.harvard.edu/, accessed on 30 November 2020). Pre-processing included intensity normalization, removal of non-brain tissue, transformation to Talairach space, segmentation of gray-white matter tissue, tessellation, and smoothing of the white matter boundary. White matter surfaces were then deformed toward the gray matter boundary at each vertex [45]. Cortical thickness was calculated based on the distance between white and gray matter boundaries at each vertex. The cortical thickness was then parcellated into 68 cortical regions (34 per hemisphere) based on the Desikan–Killiany parcellation scheme [46]. Finally, the mean thickness was extracted for each of the 68 cortical regions, as well as the mean thickness over both hemispheres. The entire cortex of each study subject was visually inspected in order to exclude regions with inaccuracies in segmentation.

### 2.4. Thickness-Based Covariance Matrices and Thresholding

A 68 × 68 Pearson’s correlation matrix of thickness measures of each parcellated brain region adjusted for overall mean thickness was used to create a binary adjacency matrix for each group (DSZ, SZND, and HC), using threshold values for the correlation coefficients. This approach depends on the assumption that positive correlations between morphometric parameters of different brain regions indicate connectivity [47]. The constructed binary adjacency matrices were composed of elements containing values of 1 (indicating connected pair of nodes) when the correlation coefficients were above the current threshold, or values of 0 in the opposite case (indicating an unconnected pair of nodes). The diagonal elements of the resultant matrices were set to zero.

**Figure 1:**
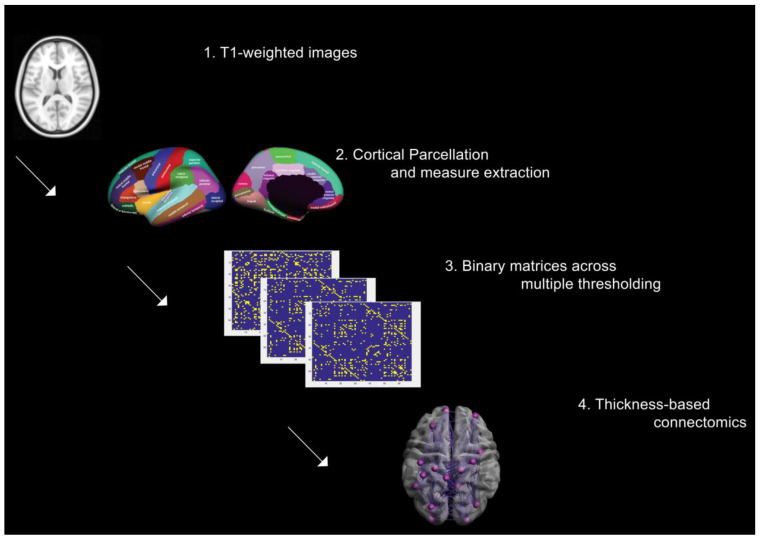
Processing steps for thickness-based networks.

Instead of choosing a single coefficient threshold, we used a range of thresholds determined by values of connection densities [48] (proportions of connections present in a graph to all possible connections). Specifically, the lower threshold was computed for each group as the minimum density to obtain a fully connected and non-fragmented network, while the upper threshold was set to 0.5 since graphs approached a random configuration beyond this density. The range of densities was increased in steps of 0.05, in order to compare the properties of emerging networks. The use of multiple threshold methods is preferred since thresholding the adjacency matrices of different groups at an absolute threshold results in networks with a different number of nodes (and degrees) that might influence the network measures, and reduce the interpretation of results across groups [49] (Figure 1).

### 2.5. Global and Local Network Measures

The relationship among brain regions within a network can be described by three groups of topological properties: *segregation* (e.g., clustering coefficient, *CC*), *integration* (e.g., characteristic path length, *L* and efficiency, *E*), and *centrality* (e.g., degree, *D*) [22,50,51] and can be quantified at either regional or global network level. *Segregation*: at the regional level, the *CC* of a node (*CC_node_*) is the number of its current links divided by the number of all possible links among its neighbors. The highest *CC_node_* has the highest localized covariance among segregated nodes of the cerebral network. The average of *CC_node_* of each region (or node) provides the global *CC* of the network (*CC_glob_*) [22]. *Integration*: *L* of a network is the average shortest path length between all pairs of nodes in the network and is the most commonly used measure of network integration. *E* is inversely related to *L*, but is numerically easier to use to estimate topological distances between elements of disconnected graphs. Specifically, *E* is a measure of the efficiency of the information transfer flow, either within segregated subgraphs or neighborhood nodes (*E_loc_*) or among the global network (*E_glob_*) [22]. *Centrality*: the *D* of a node is the number of connections that link it to the rest of the network. This is the most fundamental and readily interpretable measure of centrality for structural networks and most other measures are ultimately linked to node’s degree [22].

The topological architecture of structural networks constructed from morphometric correlations of cortical thickness data [52,53,54] has been shown to follow small-world characteristics in healthy individuals. Small-worldness of a complex network is described by two key segregation (*CC_glob_*) and integration (*L*) metrics. Thus, we computed segregation and integration indices at both regional and global network levels, in order to compare small-world indices (*Sigma*) among the three groups. In contrast, centrality metrics were computed only at the regional level, since they are less useful for global network characterization.

All topological properties were computed using Graph Analysis Toolbox (GAT) [55] which uses computation algorithms from Brain Connectivity Toolbox (https://sites.google.com/site/bctnet/, accessed on 30 November 2020).

### 2.6. Statistics

ANOVA, Student’s *t*-test, and chi-square test were performed to assess differences between groups in demographic and clinical variables using SPSS Statistics version 23.0 (IBM, Armonk, NY, USA) and considering *p* < 0.05 as the statistical threshold for significance.

To test the statistical significance of the between-group differences in brain network topology and regional network measures, nonparametric permutation tests, with 1000 repetitions, were used. In each repetition, the regional data of each participant were randomly reassigned to one of the two groups, so that each randomized group had the same number of subjects and nodes as the original groups. Then, an association matrix was obtained for each randomized group. The binary adjacency matrices were then estimated by applying the same thresholding procedure as described above. Differences in brain topological measures between the random groups were computed across the entire densities range, resulting in a permutation distribution of the difference under the null hypothesis. The actual between-group difference in network measures was then placed in the corresponding permutation distribution and a two-tailed *p*-value was calculated based on its percentile position [55]. This nonparametric permutation test based on functional data analysis (FDA) [56,57] inherently accounts for multiple comparisons across the densities range [27,58,59].

All network-based comparisons were performed by pairwise comparisons (i.e., HC vs. SDZ; HC vs. NDSZ; SZD vs. NDSZ), using GAT [55] and considering a *p_FDA_* < 0.05 as the corrected threshold for statistical significance.

## 3. Results

As expected from the matching procedure, the three groups did not differ in age and sex distribution. As for education, HC accomplished more years of formal education than both SZD and SZND groups; however, the two SZ groups did not differ between them (Table 1). SZD and SZND did not differ in illness duration, equivalent dosages of chlorpromazine, and whole-brain mean thickness. As expected, SZD suffered from more severe negative, but not positive symptoms, compared to SZND (Table 1).

### 3.1. Graph Matrices

The estimated minimum densities (*D_min_*) at which no individual brain network is fragmented, were *D_min_* = 0.17 for HC, *D_min_* = 0.11 for SZND, and *D_min_* = 0.09 for SZD groups, suggesting the need to use different lower thresholds (*D_min_*) when calculating the densities range for pairwise comparisons. Then, pairwise comparisons were performed on binary matrices, computed across a range of densities, considering the lower bound as the *D_min_* in which the networks of both groups are not fragmented (i.e., densities range: from 0.17 to 0.47 for both HC vs. SZND contrast and HC vs. SZD contrast; and from 0.11 to 0.46 for SZD vs. SZND contrast).

### 3.2. Global and Local Network Measures

Across the computed densities, HC, SZD, and SZND brain networks all showed preserved small-world indices and were not different among each other in pairwise comparisons. However, segregation measures (see 2.5 for measures description) of SZD (*CC_glob_* = 0.46) were significantly lower as compared to HC (*CC_glob_* = 0.54) (*p_FDA_* = 0.03), while integration measures did not show significant differences. No additional differences were found in segregation and integration measures comparing HC vs. SZND and SZD vs. SZND.

**Figure 2:**
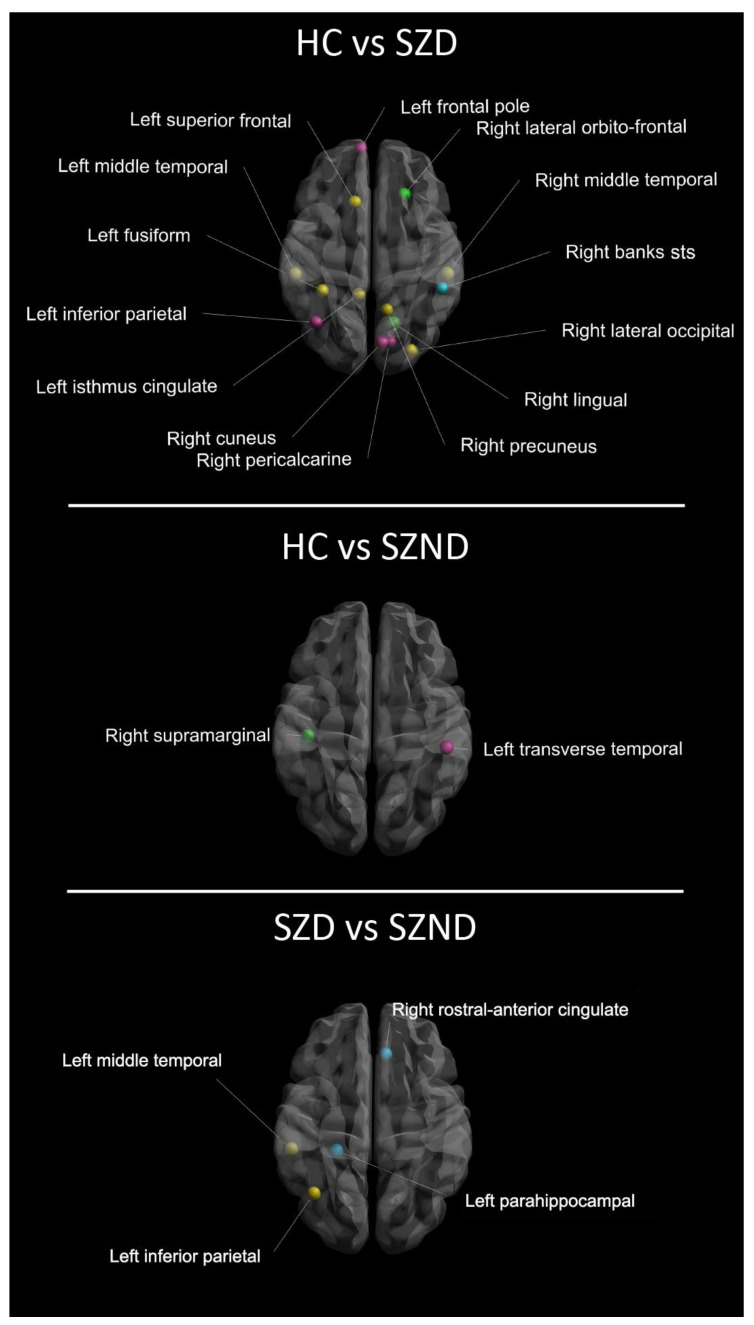
Local brain network differences from pairwise comparisons between HC and SZD, HC and SZND, and SZD and SZND. Yellow dots: brain nodes resulted as different for centrality index; green dots: brain nodes resulted as different for segregation index; blue dots: brain nodes resulted as different for integration index; purple dots: brain nodes resulted as different for multiple indices. HC, Healthy Controls; SZD, Deficit Schizophrenia; SZND, Non-Deficit Schizophrenia.

At the local level (see Table 2), comparisons between HC and SZD, at the FDA-corrected threshold, showed significant differences in all the investigated centrality, segregation, and integration measures. Indeed, SZD resulted in eight cerebral regions of abnormal *D* centrality, six regions of abnormal *CC_node_*, and five regions of abnormal *E_loc_*. Specifically, SZD showed decreased *D* centrality values in nodes of the left (inferior parietal, isthmus cingulate, and superior frontal cortices) and right (lateral occipital and pre-cuneus cortices) hemispheres. Additionally, SZD showed increased *D* centrality in the bilateral middle temporal gyri and in the left fusiform gyrus (Table 2). *CC_node_* of SZD showed reduced values in the left inferior parietal cortex and in three contiguous cortices of the right hemisphere (i.e., the cuneus, the lingual, and the pericalcarine nodes). Moreover, SZD showed increased *CC_node_* in bilateral regions of the frontal lobe (i.e., the left frontal pole and the right lateral orbitofrontal cortex) (Table 2). The *E_loc_* of SZD resulted reduced in the left inferior parietal, in the right cuneus, and pericalcarine cortices while was increased in the left frontal pole and the right banks (Table 2, Figure 2).

Differences between HC and SZND in FDA comparison were found in two regions for segregation (*CC_node_*) and one region for integration (*E_loc_*) measures. Specifically, SZND showed decreased *CC_node_* and *E_loc_* in the left transverse temporal gyrus and increased *CC_node_* in the right supramarginal gyrus (Table 2).

Comparisons between SZD and SZND showed significant FDA differencies in centrality and integration node measures. Specifically, compared to SZND, the SZD group showed decreased *D* centrality of the left inferior parietal cortex and increased *D* centrality of the left middle-temporal gyrus, in line with results found comparing SZD and HC. Moreover, SZD showed decreased *E_loc_* in the para hippocampal and increased *E_loc_* in the rostral anterior cingulate cortices (Table 2).

## 4. Discussion

Here, we investigated for the first time the connectome (brain global and local network metrics) in SZD, SZND, and HC and found three main results: (i) at the global network level only SZD differed from HC; (ii) at the local network level both SZD and SZND were different from HC, although showed alterations that were specific for each group; (iii) compared among each other at the local node level, SZD and SZND showed differences in centrality and integrational measures in nodes located in the left temporoparietal cortex and in the limbic system.

Specifically, our results showed that, compared to HC, only SZD showed a less segregated architecture of the brain global network (*CC_glob_*). Although lower *CC_glob_* in a preserved “small-world” network topology was already shown in an undifferentiated SZ sample [60], here we found that such abnormality could be referred exclusively to the SZD subgroup. The less segregated topology indicates subtle randomization in the underlying network architecture, implying a greater number of disparate cerebral clusters and dispersive links arrangement among them. This difference between SZD and HC in network clustering was also confirmed at the local level of brain nodes. Indeed, widespread nodes of the right medial occipital (cuneus, pericalcarine, and lingual) cortices as well as the left inferior parietal cortex revealed a lower *CC_node_* in SZD vs. HC. Additionally, SZD showed an increased *CC_node_* in two bilateral frontal nodes vs. HC. Further differences between SZD and HC were found for integrational and centrality network measures, in multiple fronto-parietal-temporal-limbic nodes. All these regions encompass nodes belonging either to the default mode network or to the executive control network, which are both affected in SZ as highlighted in functional connectivity studies [61,62,63]. Here, we demonstrate that both brain network systems also have structural alterations, particularly characterizing the SZD group, when compared with HC.

Unlike SZD, SZND showed more focal abnormalities when compared to HC, affecting only local nodes. Moreover, the number of affected nodes was quite low in SZND, indicating that such abnormalities are less extensive than in SZD. Specifically, SZND showed an increased segregation of the right supramarginal gyrus and a decreased segregation and integration of the left transverse temporal node (a district belonging to the superior temporal gyrus). Thus, the right supramarginal gyrus was abnormally hyperconnected only with neighbor nodes, while the left transverse temporal area was connected to more randomly spread cortical areas, affecting its efficiency in local information flow. Therefore, previous evidence of cortical abnormalities located in these brain regions in SZ as a whole [64] could be specifically referred to SZND patients and related to abnormal structural network characteristics.

Another intriguing result is that SZD showed significant differences when compared to both SZND and HC in the left inferior parietal cortex and the left middle temporal gyrus. Compared to HC, SZD showed the left inferior parietal cortex as less connected to other brain regions (lower *D*), even to their neighbors (lower *CC_node_*), resulting in a less efficient information transfer (lower *E_loc_*). The inferior parietal cortex is a multimodal association area intriguingly involved in different cognitive functions [65,66,67,68,69], such as bottom-up attention, lower-order self-perception, undirected thinking, episodic memory, and social cognition, and such functions are notoriously impaired in SZD [5]. Moreover, several pieces of evidence showed that this area is functionally impaired in undifferentiated samples of SZ (see Torrey, 2007 for a review), being associated with negative symptoms in a functional MRI study [70], and structurally affected in a volumetric 6-year follow-up longitudinal study [71] with adolescents at risk of psychosis. The association between negative symptoms and inferior parietal cortex abnormalities perfectly fits with our result showing a lower degree of centrality of this node in SZD compared to SZND. As for the left middle temporal gyrus, a previous morphometric study found that it is specifically altered in SZD [72]. Our result of increased *CC_node_* in SZD, as compared to both HC and SZND, strengthens this finding [72] and suggests that its aberrant hyper-segregated connectivity may additionally characterizes the pathophysiological substrate of SZD.

Finally, integrational differences between SZD and SZND were found in the left parahippocampal and the right anterior cingulate cortices. These structures are key components of the limbic system and are involved in negative symptoms related to motivation and goal-directed behaviors [71]. Specifically, restricted affect, curbing of interests, diminished emotional range, sense of purpose, and social drive are major negative symptoms considered to be primary in SZD. All these emotional/motivational functions are modulated through the limbic system [73] and our results intriguingly suggest that differences between SZD and SZND symptomatology could be related to the aberrant network architecture of cortices belonging to such systems.

Taken together, our results indicate that impairments in network topology involving the left inferior parietal, the middle temporal, the parahippocampal, and the right anterior cingulate cortices could be considered a neural marker that can be used to differentiate SZD from SZND.

The low prevalence of SZD and the elevated time and competencies required for the specific diagnostic process discourage many investigators and drug companies from investing more resources in this research area. Moreover, cognitive deficits and negative symptoms typical of SZD may attract less clinical attention than positive symptoms, but they are responsible for much of the morbidity associated with the disorder [74]. Within such a scenario, our study confirms the strong need to differentiate SZD from SZND and to pursue personalized treatment for each subtype.

### 4.1. Limitations

A potential weakness of the present study, linked to the low prevalence of SZD, is the relatively small sample size of the included groups. However, the groups were homogeneous with respect to clinical and demographic characteristics, reducing the potential effect of confounding variables and increasing the statistical power of comparisons. Additionally, we employed the permutation tests for the purpose of group comparisons, which are less affected by sample size and other confounding effects than the parametric one.

### 4.2. Conclusions

Our results highlighted that SZD patients are characterized by brain network abnormalities that differ from those of SZND patients. Topological differences found in network architecture between these two subtypes of SZ involve brain regions that appear to be intimately associated with the large spectrum of negative symptoms, helping to better define the symptomatic picture of SZD.

## Figures and Tables

**Table 1:** Sociodemographic and clinical characteristics of 21 HC, 21 SZND, and 21 DSZ.

	HC (21)	SZND (21)	SZD (21)	Chi, t or F	df	*p*
Gender, male (%)	17 (81)	17 (81)	17 (81)	0	2	1
Age, mean (sd)	40 (11.5)	39.95 (11.4)	39.86 (11.6)	0.001	(2;60)	0.999
Educational level, mean (sd)	15.1 (2.5)	11.48 (3.4)	11.86 (2.8)	9.739	(2;60)	0.0002 *
Mean Thick, mean (sd)	2.36 (0.1)	2.32 (0.12)	2.29 (0.1)	1.914	(2;60)	0.156
Chlorp. Eq, mean (sd)	-	338.5 (302.5)	484.3 (900.5)	−0.703	40	0.486
Illness Duration, mean (sd)	-	18.62 (12.1)	17.45 (9.5)	0.348	40	0.73
SAPS Tot, mean (sd)	-	30 (14.2)	35.2 (18.2)	−0.992	37	0.327
SANS Tot, mean (sd)	-	26.37 (12.8)	44.6 (17)	−3.772	37	0.001 *

df, degrees of freedom; sd, standard deviation; Chlorp. Eq, Chlorpromazine Equivalent of patients’ pharmacological treatment; SAPS Tot, total score of the Scale for the Assessment of Positive Symptoms; SANS Tot, total score of the Scale for the Assessment of Negative Symptoms. * Statistically significant differences at *p* < 0.05.

**Table 2:** Pairwise comparisons for regional level measures in SZD, SZND, and HC.

HC vs. SZD				HC vs. SZND				SZD vs. SZND			
Cortical Node	Avg across Densities		*p _FDA_*	Cortical Node	Avg across Densities		*p _FDA_*	Cortical Node	Avg across Densities		*p _FDA_*
	HC	SZD			HC	SZND			SZD	SZND	
***Centrality (Degree)***											
L fusiform	16	25	0.021					L inferior parietal	17	26	0.022
L inferior parietal	30	20	0.020					L middle temporal	23	14	0.018
L isthmus cingulate	26	16	0.044								
L middle temporal	16	27	0.046								
L superior frontal	30	24	0.040								
R lateral occipital	28	18	0.024								
R middle temporal	16	23	0.044								
R precuneus	29	20	0.036								
***Segregation (Clustering)***											
L inferior parietal	0.60	0.38	0.045	L transverse temporal	0.6	0.35	0.04				
L frontal pole	0.35	0.48	0.034	R supramarginal	0.56	0.75	0.04				
R cuneus	0.70	0.46	0.038								
R lateral orbito frontal	0.43	0.50	0.034								
R lingual	0.65	0.38	0.025								
R pericalcarine	0.64	0.36	0.013								
***Integration (Local Efficiency)***											
L inferior parietal	0.79	0.61	0.015	L transverse temporal	0.77	0.53	0.03	L para hippocampal	0.43	0.70	0.02
L frontal pole	0.59	0.73	0.028					R rostral anterior cingulate	0.76	0.50	0.045
R banks	0.63	0.74	0.025								
R cuneus	0.85	0.70	0.045								
R pericalcarine	0.82	0.57	0.001								

Avg = average measure; FDA = Functional Data Analysis; L = left; R = right.

## Data Availability

The dataset analyzed in the current study as well as scripting and plotting code are available from the corresponding author via email on request.
